# Regulation of the somatotropic axis by MYC-mediated miRNA repression

**DOI:** 10.3389/fcell.2023.1269860

**Published:** 2023-10-16

**Authors:** Anna P. Petrashen, Andrew D. Verdesca, Jill A. Kreiling, John M. Sedivy

**Affiliations:** Center on the Biology of Aging, Department of Molecular Biology, Cell Biology and Biochemistry, Brown University, Providence, RI, United States

**Keywords:** MYC proto-oncogene, miRNA regulation, IGF1 signaling, somatotrophic axis, gender effects, osteoporosis

## Abstract

The transcription factor MYC is overexpressed in many human cancers and has a significant causal role in tumor incidence and progression. In contrast, *Myc* ^+/−^ heterozygous mice, which have decreased MYC expression, exhibit a 10–20% increase in lifespan and a decreased incidence or progression of several age-related diseases. *Myc* heterozygous mice were also reported to have decreased mTOR and IGF1 signaling, two pathways whose reduced activity is associated with longevity in diverse species. Given MYC’s downstream role in these pathways, the downregulation of mTOR and IGF1 signaling in *Myc* heterozygotes suggests the presence of feedback loops within this regulatory network. In this communication we provide further evidence that the reduction of *Myc* expression in *Myc* ^+/−^ heterozygous mice provokes a female-specific decrease in circulating IGF1 as well as a reduction of IGF1 protein in the liver. In particular, reduced *Myc* expression led to upregulation of miRNAs that target the *Igf1* transcript, thereby inhibiting its translation and leading to decreased IGF1 protein levels. Using Argonaute (AGO)-CLIP-sequencing we found enrichment of AGO binding in the *Igf1* transcript at the target sites of let-7, miR-122, and miR-29 in female, but not male *Myc* heterozygotes. Upregulation of the liver-specific miR-122 in primary hepatocytes in culture and in vivo in mice resulted in significant downregulation of IGF1 protein, but not mRNA. Reduced levels of IGF1 increased GH production in the pituitary through a well-documented negative-feedback relationship. In line with this, we found that IGF1 levels in bone (where miR-122 is not expressed) were unchanged, consistent with the decreased incidence of osteoporosis in female *Myc* heterozygotes, despite decreased circulating IGF1.

## Introduction

MYC is a transcription factor that directly regulates 20%–30% of the genome, and indirectly influences many metabolic processes (Fernandez et al., 2003; Li et al., 2003; Patel et al., 2004; Dang et al., 2006). Deregulation of MYC is implicated in 60%–70% of all human cancers, including Burkitt’s lymphoma, breast cancer, osteosarcoma, and hepatocellular carcinoma (Greer et al., 2013). MYC has been proposed to act as a master regulator of metabolism, cell growth, and cell division (Miller et al., 2012). MYC thus appears to be a central point of metabolic regulation, integrating intrinsic growth factor signals with nutrient signals from the environment in order to determine whether the cell should grow, divide, differentiate, or undergo apoptosis (Grandori et al., 2000).

While upregulation of MYC has been extensively implicated in the context of cancer, downregulation of MYC is associated with increased health span and lifespan (Greer et al., 2013; Hofmann et al., 2015). Homozygous deletion of MYC is embryonic lethal; however, *Myc* heterozygous (*Myc* ^*+/−*^) mice show a 10% lifespan extension in males, and 20% in females (Hofmann et al., 2015). These findings are in agreement with other lifespan extending interventions which have shown that reduction of translation, energy production, oxidative phosphorylation, and ribosome biogenesis, all of which are under positive regulation by MYC, extend lifespan (Brown et al., 2008; Gems and Patridge, 2013; Johnson et al., 2013). Furthermore, upregulation of MYC results in increased generation of reactive oxygen species (ROS) and DNA damage, which are both associated with aging (Vafa et al., 2002; Hoeijmakers, 2009). Together, this large body of evidence indicates that MYC upregulation promotes cancer and aging, while downregulation promotes healthy aging and increased lifespan.

*Myc* ^*+/−*^ mice are 10%–20% smaller than their wild-type siblings, but show no changes in developmental timing or reproductive ability despite an approximately 50% reduction in MYC levels across all analyzed tissues (Hofmann et al., 2015). M*yc* ^*+/−*^ mice have increased health span, evidenced by significant amelioration of age-related phenotypes such as cardiac fibrosis, bone density loss, dysregulation of lipid metabolism and immunosenescence, and increased rotarod performance. They also display significantly higher metabolic rates and activity at both young and old ages (Hofmann et al., 2015). These observations indicate a strong impact of decreased MYC activity on age-regulated pathways.

Interestingly, liver gene expression patterns in *Myc* ^*+/−*^ mice do not overlap strongly with other life-extending interventions such as caloric restriction, resveratrol, and metformin (Hofmann et al., 2015; Ma and Gladyshev, 2017). However, several age-associated pathways are downregulated in *Myc* ^*+/−*^ mice, including insulin-like growth factor 1 (IGF1), protein kinase B (AKT), and mechanistic target of rapamycin (mTOR) signaling pathways. While these pathways are canonically upstream of MYC activity, we recently showed that MYC regulates mTOR activity by modulating glutamine uptake through direct transcriptional regulation of the amino acid transporters *Slc1a5* and *Slc7a5*, suggesting that negative feedback loops are present within these systems (Zhao et al., 2019).

IGF1 is a downstream effector of the somatotropic axis which regulates organismal growth and development in response to environmental clues such as nutrient availability, sleep, daylight, and exercise through modulation of growth hormone levels (Kato et al., 2002). IGF1 modulates somatic growth and cellular proliferation through both endocrine and autocrine/paracrine effects, with most of the endocrine-functioning hormone produced in the liver in response to growth hormone stimulation (Sullivan et al., 2002). IGF1 produced in the liver is secreted into the serum where it is found in circulation in a complex with one of seven IGF binding proteins (IGFBP1-7) and the acid-label subunit (ALS) (Rosenfeld et al., 2000). IGF1 is also produced by other tissues in both a growth hormone dependent and independent manner, but this tissue-specific production does not contribute significantly to overall IGF1 serum levels, suggesting an alternative purpose for extrahepatic IGF1 production (Le Roith et al., 2001).

Reduced IGF1 signaling is associated with increased longevity in many animal models, including nematodes, *Drosophila*, and mice, and has been correlated with longer lifespan in humans (Kenyon et al., 1993; Clancy et al., 2001; Tatar et al., 2001; Junnila et al., 2013). Decreased IGF1 signaling is however also associated with several age-related diseases such as osteoporosis, cardiovascular disease, skeletal muscle wasting and atrophy, as well as neurological ailments such as dementia (Liu et al., 2008; Elis et al., 2011). Many of these aging-related diseases can be alleviated through administration of either growth hormone or IGF1, suggesting a causal link between IGF1 decrease and development of these diseases (Yakar and Isaksson, 2015). This seeming contradiction between decreased IGF1 being simultaneously associated with increased lifespan and increased risk of age-related diseases has not been resolved but suggests that optimal health and lifespan rely on tight regulation of IGF1.

*Myc* heterozygous mice have decreased serum IGF1 levels, are long-lived, and are resistant to the development of osteoporosis, thus presenting a unique model system to address whether reducing IGF1 signaling can increase lifespan without deleterious effects on health span. We provide evidence that this effect is caused by the upregulation of specific miRNAs, which are normally repressed by Myc, and that increased levels of these miRNAs reduce the translation of the IGF-1 mRNA.

## Methods

### Use and treatment of animals

Mice were produced and housed in a specific pathogen-free AAALAC-certified barrier facility. All females used in studies were virgins. Animals of both genotypes and the same sex were housed together. Animals were kept on a 12 h light, 12 h dark light cycle with free access to food and water. The generation of *Myc* ^*+/+*^ mice was described (Hofmann et al., 2015). Animals for all experiments were produced by mating *Myc* ^*+/−*^ males with C57BL/6NCrl females purchased from Charles River. Females were purchased at 12 weeks of age and bred immediately. No animals were lost to fighting or accidental death. Dermatitis did occur in very few of the animals but was successfully treated. 48 animals (12 *Myc* ^*+/+*^ males, 12 *Myc* ^*+/+*^ females, 12 *Myc* ^*+/−*^ males and 12 *Myc* ^*+/−*^ females) were sacrificed at approximately 4 months of age, and another 48 animals (12 *Myc* ^*+/+*^ males, 12 *Myc* ^*+/+*^ females, 12 *Myc* ^*+/−*^ males and 12 *Myc* ^*+/−*^ females) were sacrificed at approximately 24 months of age for the collection of tissue specimens, at which time they were in apparent good health.

### Harvesting of tissues

Mice were euthanized between 11 a.m. and 1 p.m. Animals were euthanized one by one prior to dissection. Animals were first anesthetized by IP injection of ketamine/xylazine. Cardiac puncture was performed, and blood was collected into tubes containing heparin. Animals were then immediately euthanized by cervical dislocation. Blood samples were centrifuged at 2,200 rpm for 10 min and plasma was collected into fresh tubes and flash frozen in liquid nitrogen. Liver, pituitary, and hypothalamus tissues were quickly dissected, and flash frozen in liquid nitrogen. Soft tissue was removed from femurs and tibia, the bones were cut crosswise, and marrow was removed by centrifugation. Marrow and bone tissue were flash frozen separately. The entire dissection of each mouse was performed in under 10 min by several trained staff members working in concert on one mouse. All flash-frozen samples were subsequently stored at −80°C.

### Cell lines and culture conditions

AML-12 cells were cultured under normoxic conditions (air supplemented with 5% CO2), in a 1:1 mixture of Dulbecco’s modified Eagle’s medium (DMEM) (Hyclone, SH30243.01) and Ham’s Nutrient Mixture F12 (Hyclone, SH30026.01), supplemented with 10% FBS (Hyclone, SH30071.03), ITS supplement containing 0.005 mg/mL insulin, 0.005 mg/mL transferrin, 5 ng/mL selenium (Corning, 354350), and 40 ng/mL dexamethasone (MP Biomedicals, 0219456125).

### Hepatocyte isolation, growth hormone stimulation, and transfection

Hepatocyte isolation was performed using a two-step perfusion method as previously described with some modification (Klaunig et al., 1981). Mice were anesthetized using IP injection of ketamine/xylazine mixture as described in section 2.4. Perfusion was done through cannulation of the inferior vena cava with drainage through the portal vein. First, approximately 40 mL at a flow rate of 6 mL/min of HBSS with 0.5 mM EGTA (without calcium or magnesium) was perfused to flush the liver. Second, 40 mL of digestion media (low-glucose DMEM with 200 mg/mL calcium, 20 mM HEPES, and 80 U/mL collagenase IV (Worthington)) was perfused until liver was digested. Liver was then excised, and the cells liberated from the capsule through gentle mincing. Hepatocytes were then filtered through a 70 µm filter and washed three times with cold isolation media (high-glucose DMEM with 10% FBS, pen/strep, 200 mM glutamine). Cell viability and number was assessed with Trypan blue staining, and cells were plated at 600,000 cells/well in 6-well Primaria plates (Corning). After a 2 h incubation to allow cells to attach, media was replaced with culture media (low-glucose DMEM, pen/step, 200 mM glutamine, insulin, transferrin, selenium, dexamethasone, epidermal growth factor) with or without transfection reagents. Transfection was carried out using Fugene HD according to manufacturer’s protocol with a 4:1 ratio of DNA to reagent in RNase and DNase-free sterile water using a 10 min incubation time to allow for the formation of complexes prior to addition into cell media. miRNA miRcury mimics (Qiagen) were added at 20 nM concentration with DNA carrier for a total DNA concentration of 1 ug. After 4 h of incubation with transfection reagents, media was replaced with culture media to reduce cytotoxicity and cells were incubated for 24 h prior to harvesting. Growth hormone stimulation (where performed) was carried out in the final 2 hours or incubation by replacement of media with culture media containing 50 nM mouse recombinant growth hormone. For harvesting, cells were washed twice with ice-cold PBS, then lifted with a cell scraper and pelleted by centrifugation at max speed for 2 minutes. Cell pellets were stored at −80°C.

### Preparation of RNA

20–50 mg fragments of tissue were removed from −80°C, weighed, and homogenized in 1 mL Trizol reagent (Invitrogen) using a Fisher PowerGen 125 motorized homogenizer at room temperature. 200 μL chloroform was added, the samples were vortexed, and incubated at room temperature for 2–3 min (as per manufacturer’s protocol). Samples were then centrifuged at 12,000 x G for 15 min, and the resulting aqueous layer was further purified using the RNeasy Mini Kit (Qiagen) according to manufacturer’s instructions. RNA quality and concentration was accessed using a NanoDrop 2000 spectrophotometer. For RNA used in RNA-Sequencing experiments, RNA quality was further accessed using an Agilent 2100 Bioanalyzer. Only samples with a RIN of greater than 9 were used in sequencing experiments.

### RT-qPCR

1 µg of RNA was reverse transcribed into cDNA in 50 µL reactions using the Taqman kit (Applied Biosystems), according to the manufacturer’s protocol. 1 μL of this reaction was used in subsequent qPCR reactions for the assessment of mRNA abundance, which were performed using the SYBR Green system (Applied Biosystems) on the ABI 7900 Fast Sequence Detection instrument, according to manufacturer’s specifications. All primer sequences are listed in Supplementary Table S1. mRNA expression was normalized to GAPDH (primer pair 4) and verified using beta actin (primer pair 5) and Beta-2 microglobulin (primer pair 6). miRNA RT-qPCR was performed as described previously (Busk *2011* BMC Biotechnology). Briefly, 500 ng of RNA was reverse transcribed into cDNA in 50 µL reactions containing 5 µL 10x Poly(A) polymerase buffer, 0.1 mM ATP, 1 µM of RT primer (was 5′-CAGGTCCAGTTTTTTTTTTTTTTTVN, where V is A, C and G and N is A, C, G and T.), 0.1 mM of each deoxynucleotide (dATP, dCTP, dGTP, and dTTP), 500 units MuLV reverse transcriptase (New England Biolabs), and five units of poly(A) polymerase (New England Biolabs). The reaction was incubated for 1 h at 42°C, followed by enzyme inactivation at 95°C for 5 min qPCR was performed using the Sybr Green system as above. Snord 70 (primer pair 42) was used for normalization.

### Assessment of miRNA abundance using the nanostring platform

Total RNA was isolated as above and diluted to a concentration of 33.3 ng/uL. 3 uL of each sample was run on the Nanostring platform using the nCounter Mouse v1.5 miRNA panel according to manufacturer’s instructions.

### Protein extraction for enzyme-linked immunosorbent assays

Liver and pituitary protein extracts were prepared by homogenizing 5–10 mg of tissue in 60 μL/mg of tissue extraction buffer containing 100 mM Tris (pH 7.4), 150 mM NaCl, 1 mM EGTA, 1 mM EDTA, 1% Triton X-100, 0.5% sodium deoxycholate, 1 mM phenylmethylsulfonyl fluoride (PMSF), and 1X cOmplete mini protease inhibitor cocktail (Sigma-Aldrich 11836153001). Samples were homogenized using either the Fisher PowerGen 125 motorized homogenizer, or by passage through a 26 G needle. Homogenized extracts were incubated on ice for 20 min, then centrifuged at maximum speed for 10 min at 4°C in a microcentrifuge. The resulting supernatant was diluted 1:20 with MilliQ water prior to assessment of concentration using the Qubit Protein Assay kit (Qiagen Q33211).

### Immunoblotting

Liver protein extracts used for immunoblotting were prepared by homogenizing 30–50 mg of tissue in 1 mL laemmli sample buffer (60 mM Tris (pH 6.8), 2% SDS, .05% bromphenol blue, 10% glycerol, 100 mM DTT, and 1X cOmplete mini protease inhibitor cocktail (Sigma-Aldrich 11836153001)). Samples were homogenized using the Fisher PowerGen 125 motorized homogenizer, then boiled for 5 min, cooled, and centrifuged at maximum speed for 10 min at 4°C. Protein concentration in the resulting supernatant was quantified using the Qubit Protein Assay kit (Qiagen Q33211), samples were diluted to 10 μg/μL protein, and stored at −80°C. For the assessment of IGF binding protein abundance, samples were boiled for 5 min, then run at 100 µg protein/well on 15% polyacrylamide gels. Gels were transferred to low-fluorescence PVDF membrane (Invitrogen, 22860). Membranes were blocked in PBS containing 5% BSA with 0.2% Tween-20, then stained with the following primary antibodies; ribosomal protein S6, S6K (Cell Signaling Technologies #2317), insulin-like growth factor binding protein 3, IGFBP3 (Santa Cruz sc-9028), enhancer of zeste homologue 2, EZH2 (Cell Signaling Technologies # 5246), and glyceraldehyde-3-phosphate dehydrogenase GAPDH (Millipore #G8795).

### Quantification of IGF1 protein by enzyme-linked immunosorbent assay

**FIGURE 1:**
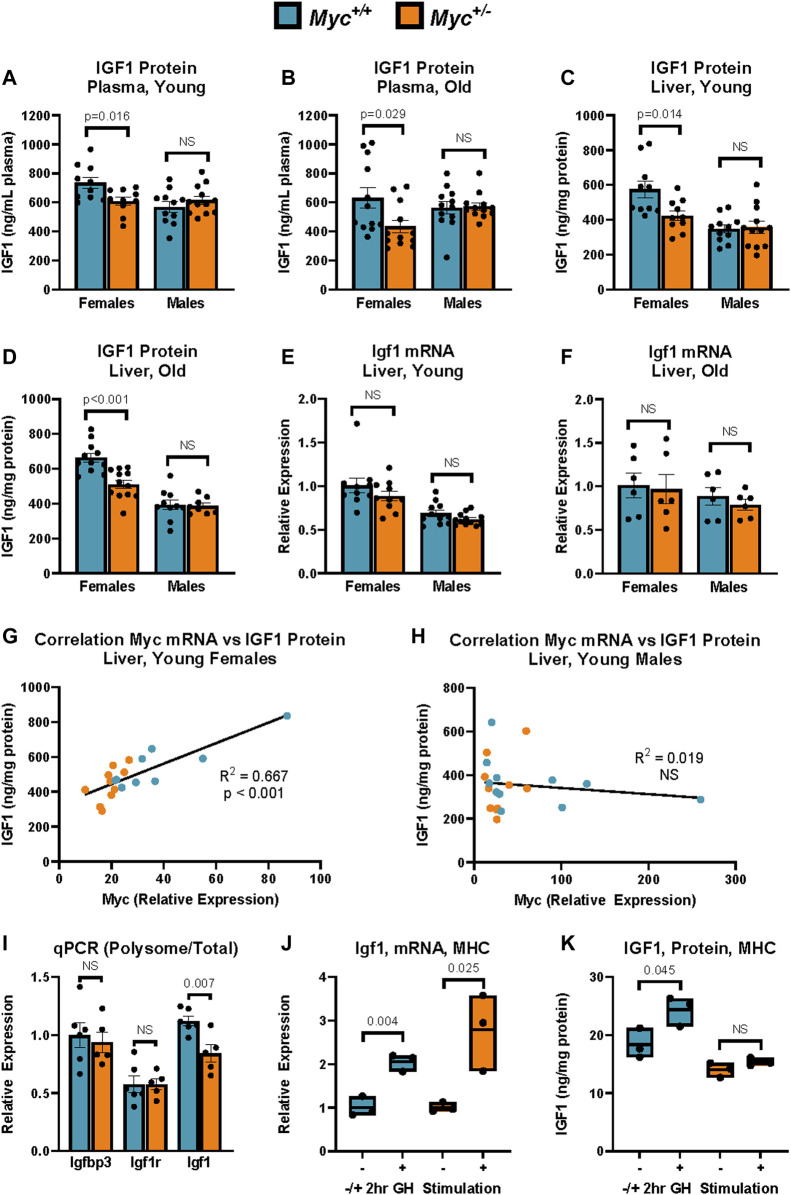
IGF-1 Expression in *Myc* ^*+/+*^ and *Myc* ^*+/−*^ mice at young and old ages. **(A)** Total plasma IGF1 protein levels as assayed by ELISA. *n* = 10–12, 3 months, males and females. **(B)** Total plasma IGF1 protein levels as assayed by ELISA. n = 10–12, 24 months, males and females. **(C)** Total liver IGF1 protein levels as assayed by ELISA. n = 10–12, 3 months, males and females. **(D)** Total liver IGF1 protein levels as assayed by ELISA. n = 10–12, 24 months, males and females. **(E)** *Igf1* mRNA levels (primer pair 7) as measured by RT-qPCR in liver. n = 10–12, 3 months, males and females. **(F)** *Igf1* mRNA levels (primer pair 7) as measured by RT-qPCR in liver. n = 10–12, 24 months, males and females. **(G, H)** *Myc* mRNA levels (primer pair 3) as measured by RT-qPCR relative to corresponding liver IGF1 protein levels assessed as in **(B)** in females **(G)** and males **(H)**. *n* = 9–11, 3 months. **(I)** Polysome-associated mRNA was isolated by sucrose-gradient centrifugation. Transcript abundance in the polysome-bound mRNA fraction was quantified by RT-qPCR for *Igfbp3* (primer pair 5), *Igf1r* (primer pair 6), and *Igf1* (primer pair 7), and expressed as fraction of transcript abundance in total mRNA, relative to GAPDH (primer pair 4). n = five to six, females, 4 months. **(J)** Igf1 mRNA expression (primer pair 7) as measured by RT-qPCR in primary mouse hepatocytes (MHC) of *Myc* ^*+/−*^ and *Myc* ^*+/+*^ mice with and without treatment with 100 nM recombinant mouse growth hormone for 2 h *n* = 3, females, 6–8 weeks. **(K)** IGF1 protein expression as measured by ELISA in primary mouse hepatocytes (MHC) of *Myc* ^*+/−*^ and *Myc* ^*+/+*^ mice with and without treatment with 100 nM recombinant mouse growth hormone for 2 h. Normalized to total protein. *n* = 3, females, 6–8 weeks. Statistical significance was computed using Student’s t-test **(A–F and I–K)**. Correlation for **(G, H)** was computed using Pearson’s product-moment correlation sample estimate. Error bars represent SEM.

Liver and plasma total IGF1 protein abundance was quantified using the Abcam IGF1 ELISA kit (Abcam ab100695) as per manufacturer’s protocol. Liver and cell extracts (Figures 1B, E; Figures 3C, G) and plasma samples (Figures 1A, D; Figure 3I) were diluted 1:1 and 1:100, respectively, in assay buffer. All samples were run in duplicate. Absorbance readings were normalized to a standard curve generated from readings of standard solutions of known IGF1 concentration. IGF1 measurements for liver extracts were normalized to protein concentration as determined by the Qubit Protein Assay kit (Qiagen Q33211).

### Quantification of growth hormone by enzyme-linked immunosorbent assay

Pituitary and plasma growth hormone abundance was quantified using the EMD Millipore Rat/Mouse Growth Hormone ELISA kit (EMD Millipore, EZRMGH-45 K). All samples were run in duplicate. Pituitary extracts were prepared as described above and diluted 1:5000 in sample buffer for the assay. Blood was collected every 48 h for a total of three time points between 10 and 11a.m. by saphenous vein blood collection. Samples were centrifuged at 2,200 rpm for 10 min at 4°C, and plasma was removed to a clean tube. 10 uL of undiluted plasma per mouse per timepoint was used in the assay. Absorbance readings were normalized to standard curve generated from readings of standard solutions of known growth hormone concentration. Pituitary growth hormone measurements were normalized to protein concentration as determined by the Qubit Protein Assay kit (Qiagen Q33211).

### Bone density measurements

L4 vertebrae were scanned using a Scanco Medical Micro-CT 40 system to acquire approximately 250 slices per sample at 10 μm resolution. The volume containing trabecular bone (cortical bone was omitted) was selected by someone blind to the age or genotype of the mouse. The morphometric parameters of bone volume per total volume, trabecular spacing, and trabecular number, were computed for each vertebra, and the average, standard error, and *p*-value (Student’s *t*-test) were determined for each cohort.

### Polyribosome profiling

Polysome enriched fractions were obtained by dounce homogenizing 1 g of liver tissue per animal in 3 mL homogenization buffer (50 mM HEPES pH 7.4, 250 mM KCl, 5 mM MgCl_2_, 250 mM sucrose, 200 U/mL RNasin, and 1 μg/mL microcystin). Samples were cleared by centrifugation at 3,000 X G, 4°C, for 15 min. For each mL of supernatant, 100 µL of 10% Triton X-100 and 100 µL of 13% sodium deoxycholate (NaDOC) was added. Samples were loaded onto 10%–50% sucrose gradients and centrifuged at 22,500 rpm for 19 h, 9 min in a Beckman SW-28 rotor at 4°C (acceleration and deceleration set to 7). Columns were fractionated using an Isco Density Gradient Fractionator at a flow rate of 2 mL/min while absorbance at 254 nm was monitored using an Isco UA-5 Absorbance/Flourescence Detector. Fractions determined from the spectral graph to contain polyribosomes were then pooled for RNA extraction. To each sample 500 mM EDTA was added to achieve a final concentration of 20 mM, prior to incubation for 5 min at room temperature. 20% SDS was added to a final concentration of 0.5%, and samples were incubated for 10 min at room temperature. An equal volume of RNase-free water was added, followed by an equal volume of acid phenol:chloroform (Ambion, 9722). Samples were then centrifuged at 12,000 X G for 30 min at 4°C, and the aqueous layer reserved. RNA was precipitated overnight at −80°C using 2.5 volumes of 100% ethanol and 0.1 volume of 5M NH_4_Oac (Ambion, 9071), washed twice with 75% ethanol, and resuspended in water. RT-qPCR was conducted as described above and compared to RNA from unfractionated liver.

### Argonaut crosslinking immunoprecipitation followed by sequencing sample preparation

Argonaute CLIP-Sequencing libraries were prepared as previously described (Moore et al., 2014). Frozen liver samples were ground under liquid nitrogen in a mortar and pestle to a fine powder. A small fraction of powder was reserved for total RNA extraction. Ground tissue was then irradiated on dry ice at 400 mJ per cm^2^ and then again at 200 mJ per cm^2^ using a Stratlinker XL-1500 (Stratagene) UV cross-linker and stored at −80°C until further use. Cross-linked tissue was resuspended in three times volume of PXL buffer (1X PBS containing 1% Igepal/NP-40, 0.5% sodium deoxycholate, and 0.1% SDS) and incubated on ice for 10 min. Samples were then treated first with 30 µL of DNase I per mL of lysate (5 min at 37°C with agitation at 1,000 rpm) then with 10 uL per mL of lysate of 1:10,000 dilution of RNase A (5 min at 37°C with agitation at 1,000 rpm). RNase digestion was stopped with 2.5 µL per mL of lysate of RNAsin Plus. Lysates were then centrifuged at maximum speed for 20 min at 4°C. Beads for immunoprecipitation were prepared by washing 200 µL (per sample) of Dynabeads A three times in PBS with 0.02% Tween-20, incubating with 50 µg of rabbit anti-mouse IgG bridging antibody for 30 min at room temperature with end-to-end rotation, repeating the wash steps, then incubating with 4 µL anti-Ago 2A8 antibody in PBS with 0.02% Tween-20 with end-to-end rotation. Beads were then washed three times in PXL buffer prior to addition of cross-linked tissue lysates. Lysate/bead mixtures were then rotated end-to-end for 2 h at 4°C. Beads were then washed three times with cold PXL buffer, then twice with 5 PXL buffer (PXL buffer with 5X PBS), then twice with PNK buffer (50 mM Tris-HCL pH 7.5, 10 mM MgCl_2_, and 0.05% Igepal/NP40). To prepare RNA 3′ ends for linker ligation, beads were resuspended in 80 µL of dephosphorylation buffer containing 3 U of CIAP and RNAsin Plus inhibitor and incubated for 20 min at 37°C with shaking at 1,000 rpm for 15 s every 2 min. Beads were washed once with PNK buffer, once with PNK buffer plus 20 mM EGTA, then twice with PNK buffer. Radiolabeled 3′ linkers were prepared using T4 polynucleotide kinase following manufacturer’s instruction using 25 µL 32P-γ-ATP and 200 pmol of a dephosphorylated and 3′inverted ddT blocked L32 RNA linker (sequence: GUGUCAGUCACUUCCAGCGG/3InvdT/, IDT) and incubated for 30 min at 37°C. 2 μL of 1 mM ATP was added and the mixture incubated for another 5 min to drive the reaction to completion. To purify the reaction from free nucleotides, the reaction was passed through a G-25 column following manufacturer’s instructions. To ligate the radiolabeled linker, beads were resuspended with T4 RNA ligase as per manufacturer’s instruction with 12 pmol of radiolabeled linker and incubated at 16°C with shaking at 1,000 rpm for 15 s every 2 min. After 1 hour, an additional 60 pmol of unlabeled, phosphorylated linker was added and the reaction allowed to proceed overnight. Following 3′ linker ligation, beads were washed twice with PXL, twice with 5X PXL, and twice with PNK buffers. To restore the 5′ phosphate, beads were resuspended in 80 µL of T4 PNK phosphorylation mix according to manufacturer’s instruction and incubated for 20 min at 37°C with shaking at 1,000 rpm for 15 s every 2 min. The beads were then washed three times with PNK plus 20 mM EGTA buffer. To elute protein:RNA complexes, beads were resuspended in 100 µL of LDS sample buffer with 10% reducing agent, then incubated at 70°C for 10 min with constant shaking at 1,000 rpm. Samples were then loaded onto an 8% Novex NuPAGE Bis-Tris gel in SDS-MOPS buffer run at 175 V, and transferred to Protran BA-85 nitrocellulose using a Criterion blotter at 90 V in NuPAGE transfer buffer containing 10% (vol/vol) methanol. Nitrocellulose membrane was rinsed in PBS and exposed to Biomax MR film (Kodak) at −80°C overnight. Regions corresponding to 110–150 kd were then excised and RNA liberated from the nitrocellulose by incubation with 4 mg/mL proteinase K in PK buffer (100 mM Tris-HCl, pH 7.5, 50 mM NaCl, and 10 mM EDTA) for 20 min at 37°C with constant agitation at 1,000 rpm. 200 μL of 7M urea in PK buffer was added and the incubation proceeded for another 20 min. RNA was then extracted using acid phenol:chloroform and precipitated overnight at −20°C with two times volume of 1:1 ethanol:isopropanol. RNA was pelleted, washed twice with 75% ethanol, and resuspended in 6 µL water. A T4 RNA ligase 5′ ligation reaction was prepared according to manufacturer’s protocol with 20 pmol of RL5D linker (sequence:/5InvddT/AGGGAGGACGAUGCGGNNNNG, IDT) in a total volume of 10 µL and allowed to proceed overnight at 16°C. DNase digestion was then performed using RQ1 DNase according to manufacturer’s instructions with an incubation of 20 min at 37°C in a total reaction volume of 100 µL. RNA was then reprecipitated as described above. RT-PCR of was carried out using SuperScript III (Invitrogen) following the manufacturer’s instructions using the DP3 primer for reverse transcription (sequence: CCG​CTG​GAA​GTG​ACT​GAC​AC). PCR was performed immediately after RT using 27 µL Accuprime Pfx (Invitrogen), and 333 pmol each of DP3 and DP5 (sequence: AGGGAGGACGATGCGG) primers for each 2.5 µL of RT reaction. PCR conditions were 95°C for 2 min, 27 cycles of 95°C for 20 s denature, 58°C for 30 s anneal, and 68°C for 30 s extension. PCR reactions were cleaned up using PureLink Quick PCR Purification Kit (Invitrogen, K310001) and eluted in 30 µL of elution buffer. Next, sequencing adapters were added by PCR using Accuprime Pfx with 333 pmol each of TSP5 and TSP7.1-TSP7.12 primers for each 2 µL of sample. DNA was then size selected by gel purification on 10% polyacrylamide gels. Regions between 190–300 kd were excised and DNA was extracted using the Qiaquick Gel Extraction kit and eluted in 30 µL. Library quality and concentration was analyzed using an Agilent 2100 Bioanalyzer. Multiplexed sequencing was performed on a NextSeq550 High Throughput Benchtop Sequencer (Illumina) as 75 bp single-end reads with 15% PhiX spike.

### Argonaute CLIP-Seq bioinformatic analysis

Bioinformatic analysis of CLIP-Seq data was performed using the galaxy suite of bioinformatic tools (http://galaxyproject.org/ (Goecks et al., 2010)). FASTQ files were filtered for reads with quality score of 20 or greater in 80% or more base pairs. Reads were then collapsed to eliminate sequencing and PCR duplicates. Cutadapt was used to trim 3′ and 5′ linker sequences, as well as discard reads shorter than 18 nucleotides. Reads were aligned to the mm10 genome with STAR using default parameters and a permissible mismatch rate of 0.3 per read. miRNA target sites in the *Igf1* transcript were downloaded from TargetScan and reads overlapping these sites were counted. Counts at each miRNA seed sequence were determined by FeatureCounts, and differential abundance and significance was assessed by DeSeq2.

### *In vivo* miRNA delivery

For assessment of the *in vivo* effects of miRNA upregulation, 3 month-old female C57Bl/6N mice purchased from Charles River were tail vein injected with Qiagen miRcury miRNA mimics of let-7i (Catalog #YM00471739-AGA), miR-122 (Catalog #YM00470430-AGA) or scrambled control 5 (Catalog #YM00479904-AGA). Injections were prepared by combining 1 nmol of each miRNA mimic or scrambled control with invivofectamine complexation buffer prior to addition to an equal volume of invivofectamine (Catalog #IVF3005, ThermoFisher). Complexes were allowed to form by incubation for 30 min at 50°C according to manufacturer’s instructions. Complexes were then diluted with PBS to achieve a final volume of 200 µL and the full volume was injected via the tail vein. Mice were euthanized at 4 days post-injection, liver was perfused with PBS via cannulation of the IVC and snipping of the portal vein, and tissues were harvested and flash frozen as described above.

### Statistical analysis

Data are shown as means with SEM (unless stated otherwise). N indicates the number of animals per test group; age and sex are also noted.

## Results

In circulation, ∼95% of IGF1 is bound by one of seven IGF binding proteins (IGFBPs) and the acid-labile subunit (ALS) (Rosenfeld et al., 2000). This tight binding of IGF1 to IGFBPs impedes detection by antibodies used in common ELISA assays, thus additional steps are required to assess total IGF1 levels. In order to assess whether total IGF1 levels were decreased in *Myc* ^*+/−*^ mice, we prepared sample dilutions in a buffer containing an excess of IGF2, which is not expressed at significant levels in adulthood, but has equal affinity for IGFBPs. This allows for IGF2 to outcompete IGF1 in binding to the present IGFBPs, thus freeing IGF1 to allow detection by anti-IGF1 antibodies. Using this approach, we determined that consistent with previously published results (Hofmann et al., 2015), total plasma IGF1 levels were decreased in young and old female *Myc* ^*+/−*^ mice by ∼20%–30% (Figures 1A, B).

Interestingly, we observed no difference in IGF1 plasma levels in male *Myc* ^*+/−*^ mice, suggesting a sexual dimorphism in the effect of MYC on IGF1. We next analyzed *Igf1* transcript and protein levels in liver, which is the main site of synthesis of circulating IGF1 (Sjogren et al., 1999). Consistent with the plasma data, we saw an ∼20% decrease in IGF1 protein levels in young and old female *Myc* ^*+/−*^ mouse liver tissue compared to *Myc* ^*+/+*^ mice, and no significant change in male *Myc* ^*+/−*^ mouse liver (Figures 1C, D). However, *Igf1* transcript levels were unchanged in either sex in both young and old mice (Figures 1E, F). In line with this, IGF1 protein corresponded to *Myc* transcript abundance in female, but not male, mice in both *Myc* ^*+/−*^ and *Myc* ^*+/+*^ genotypes (Figures 1G, H). While we find that *Myc* transcript levels exhibit significant overlap between the two genotypes, particularly in males, our previously published data shows that *Myc* ^*+/−*^ mice exhibit an approximately 50% decrease in MYC protein levels in liver of male and female mice (Hofmann et al., 2015). Taken together, these results suggest that MYC positively regulates IGF1 protein levels post-transcriptionally in a sex-specific manner, and that decreased MYC expression in female *Myc* ^*+/−*^ mice results in lower IGF1 protein levels in the liver, which results in decreased levels of circulating IGF1.

To assess whether IGF1 translation is regulated in female *Myc* ^*+/−*^ mice, we isolated polysome-bound mRNA from liver extracts using sucrose density centrifugation. We found that while related transcripts such as *Igfbp3* and *Igf1r* showed no changes in polysome association, the *Igf1* transcript was reduced by ∼20% in the polysome-associated mRNA fraction in the *Myc* ^*+/−*^ *mice* (Figure 1I). This result suggests that MYC regulates IGF1 by inhibiting its translation. MYC is a known regulator of genes involved in translation, and *Myc* ^*+/−*^ mice do show a slight decrease in overall rates of translation (Hofmann et al., 2015). However, the lack of significant translational repression on transcripts related to and regulated by the same pathways as *Igf1,* such as its binding proteins and receptor, suggests that the regulation of *Igf1* translation by MYC is a targeted effect, rather than a global one.

To determine whether hepatocytes from female *Myc* ^*+/−*^ mice respond to growth hormone stimulation as efficiently as *Myc* ^*+/+*^ hepatocytes, mouse primary hepatocytes (MHC) were isolated using a two-step perfusion protocol and allowed to adhere for 24 h in cell culture. Hepatocytes from both genotypes were then treated with 100 nM of mouse recombinant growth hormone for 2 h prior to harvest and extraction of mRNA and protein. We found that *Igf1* transcript levels were upregulated to a similar extent in *Myc* ^*+/−*^ and *Myc* ^*+/+*^ hepatocytes (Figure 1J). However, while *Myc* ^*+/+*^ hepatocytes show a 30% increase in IGF1 protein in response to growth hormone stimulation, treatment of *Myc* ^*+/−*^ hepatocytes with growth hormone did not result in significant IGF1 upregulation (Figure 1K). Together, these data suggest that low MYC levels impede the translation of the *Igf1* transcript.

Given the known role of MYC in regulating the expression of miRNA genes, as well as the ∼6 kb length of the *Igf1* 3′UTR, we next assessed whether MYC-regulated miRNAs could target the *Igf1* transcript. TargetScan prediction identified 117 conserved miRNA binding sites in the *Igf1* 3′UTR, including those of 11 of the most expressed miRNAs (Lewis et al., 2005). 56 of these miRNAs have been previously shown to be repressed by MYC and are thus predicted to be downregulated in *Myc* ^*+/−*^ mice (Chang et al., 2008). To assess whether miRNAs known to be repressed by increased MYC levels and predicted to target the *Igf1* transcript are indeed upregulated in *Myc* ^*+/−*^ mice, we analyzed their expression in total RNA extracts from livers of 3-month-old *Myc* ^*+/−*^ and *Myc* ^*+/+*^ mice using the Nanostring platform.

**FIGURE 2:**
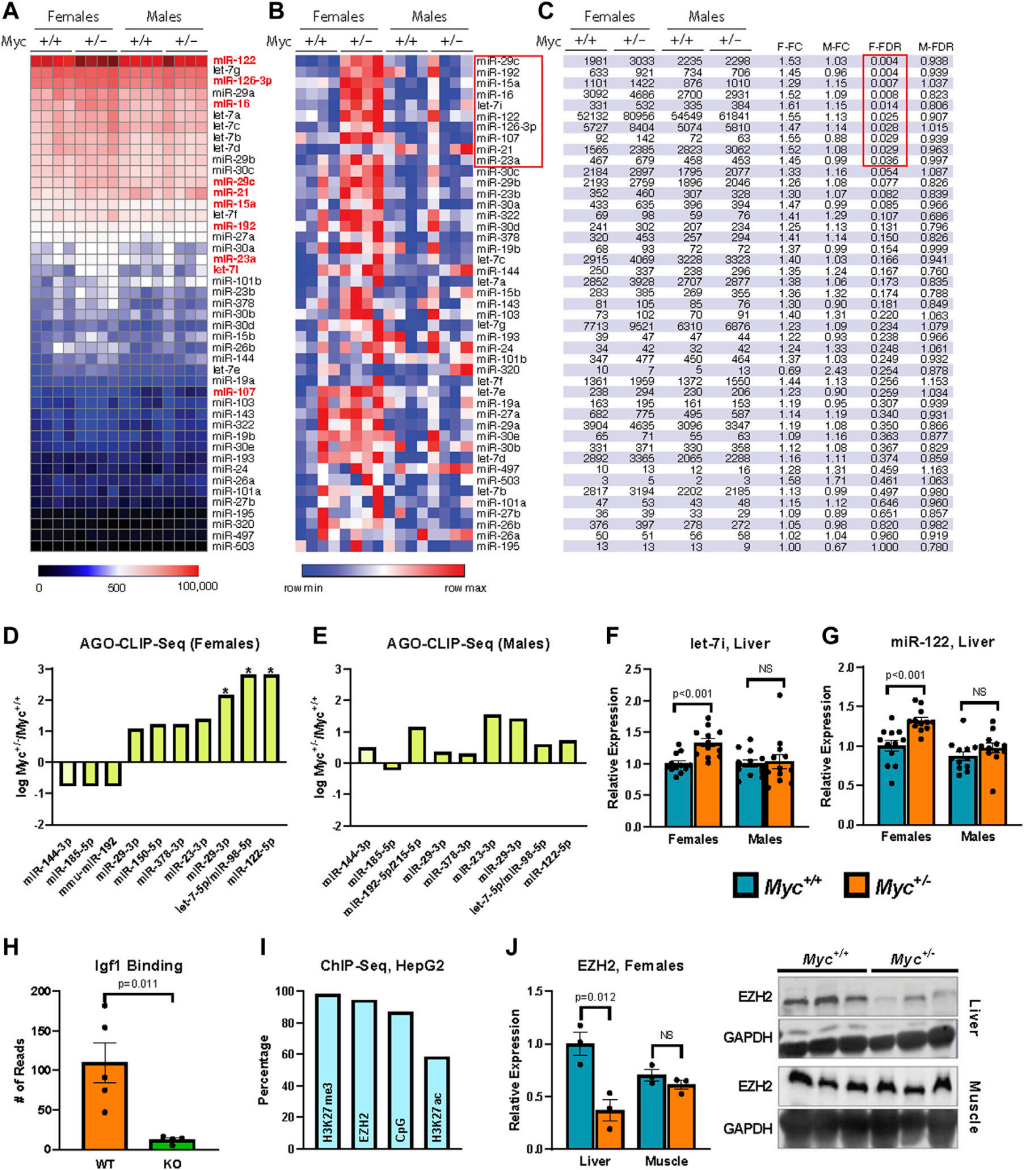
Regulation of miRNA expression by MYC **(A, B)** Heatmaps showing relative expression of candidate miRNA genes quantified by the Nanostring platform on total RNA extracted from liver of female and male mice are shown reflecting global levels of expression **(A)**, or for each miRNA across conditions (row min-max **(B)**). *n* = 4, 3 months old, males and females. **(C)** miRNA gene expression in table format showing the significant changes (FDR-corrected *p*-value <0.25). **(D, E)** Enrichment of miRNA target sequences in the *Igf1* transcript as determined by sequencing of AGO-bound RNA isolated from liver of *Myc* ^*+/−*^ and *Myc* ^*+/+*^ female **(D)** and male **(E)** mice. *n* = 3–4, 3 months old. **(F)** Expression of let-7i as determined by RT-qPCR (primer pair 11) in liver of *Myc* ^*+/−*^ and *Myc* ^*+/+*^ mice. Normalized to Snord70 (primer pair 13). *n* = 10–12, females and males, 3 months old. **(G)** Expression of miR-122 as determined by RT-qPCR (primer pair 12) in liver of *Myc* ^*+/−*^ and *Myc* ^*+/+*^ mice. Normalized to Snord70 (primer pair 13). *n* = 10–12, females and males, 3 months old. **(H)** Enrichment of miR-122 target sequence in the *Igf1* transcript in WT and miR-122 KO mouse liver, dataset originally generated by (Luna et al., 2017). *n* = four to five, females and males, 5 months old. **(I)** Percentage of candidate miRNA gene promoters (defined as ± 1,000 bp of transcription start site) with enrichment for H3K27me3, EZH2 and H3K27ac (ENCODE datasets doi:10.17989%2FENCSR000AOL, doi:10.17989%2FENCSR000ARI and doi:10.17989%2FENCSR000AMO, respectively) as well as proximity to CpG islands as determined by analysis of available ChIP-Seq datasets in HepG2 cells. **(J)** EZH2 liver and muscle protein levels were determined by immunoblot and quantified using ImageJ. Expression normalized to GAPDH. *n* = 3, 30-month old, females. The image of the immunoblot is shown to the right of the graph. Statistical significance was computed using Student’s t-test and followed by FDR correction in the case of multiple comparisons **(C–E)**. Error bars represent SEM.

Of the 56 candidate miRNAs identified above, 45 were found to have detectable expression in liver tissue of 3-month-old mice (greater than five counts in at least one sample). We found 10 candidate miRNAs to be significantly (FDR-adjusted *p*-value <0.05) upregulated in female, but not male *Myc* ^*+/−*^ mice (Figures 2A–C). Interestingly, no downregulated miRNAs were found for either sex. These results are consistent with the literature on miRNA regulation by MYC, which documents that the majority of miRNA genes are downregulated in the context of MYC overexpression (Chang et al., 2008).

In order to validate that upregulated candidate miRNAs in *Myc* ^*+/−*^ female mice lead to increased targeting of the *Igf1* transcript *in vivo*, we performed AGO CLIP-Seq on liver tissue of both genotypes and sexes (Moore et al., 2014). Briefly, flash frozen liver was pulverized and cross-linked using UV-light prior to protein extraction in the presence of RNase inhibitors. Argonaute immunoprecipitation was then carried out, and Argonaute:miRNA:mRNA complexes were isolated by gel electrophoresis using a radiolabeled 3′ linker for visualization. A 5′ linker containing a degenerate sequence was ligated to the isolated RNA tags to allow for PCR amplification and subsequent sequencing and filtering of duplicate reads. After filtering for duplicate and low-quality reads, sequences were aligned to the mm10 genome, and MACS was used to call peaks (Zhang et al., 2008). miRNA seed sequences were identified within 50 bp of peak centers using Targetscan (Lewis et al., 2005).

We found that in female *Myc* ^*+/−*^ mice, AGO binding of the *Igf1* transcript was significantly enriched at the target sites of miR-122, let-7, and to a more modest degree miR-29 (Figures 2D, E). These three miRNA families are among the most highly expressed miRNAs in the liver, and all three were found to be significantly upregulated in female *Myc* ^*+/−*^ mice in our Nanostring analysis. Given these results, we validated our Nanostring results of let-7i and miR-122 expression by RT-qPCR modified for miRNA detection (Balcells et al., 2011; Busk, 2014). We found that both let-7i and miR-122 are upregulated by ∼30% in liver of female, but not male *Myc* ^*+/−*^ mice (Figures 2F, G), consistent with our previous analysis.

miR-122 is a liver-specific miRNA that compromises ∼70% of the total miRNA species in mouse liver (Jopling, 2012). Furthermore, as decreased miR-122 expression has been linked to hepatocellular carcinoma, its effects have been investigated in the liver, and an AGO CLIP-Seq dataset is available from a miR-122 liver-specific knockout mouse model (Luna et al., 2017). We analyzed this available dataset and found that miR-122 knockout significantly reduces AGO binding at the predicted miR-122 target site in the *Igf1* transcript, thus further validating this site as a *bona fide* miR-122 target (Figure 2H). Given that our AGO-CLIP-Seq data identified the target sites for let-7 and miR-122 as the most enriched for AGO binding in female *Myc* ^*+/−*^ mouse liver, we chose these two miRNAs for further analysis.

MYC has previously been implicated as a positive regulator of the enhancer of zeste homologue 2 (EZH2) (Ito et al., 2018). EZH2 is a methyltransferase that, as part of the Polycomb Repressive Complex 2 (PRC2), di-/tri-methylates histone 3 lysine 27 (H3K27) to promote the heterochromatization of target regions (Bracken and Heln, 2009). We thus analyzed available Encode ChIP-Seq datasets from the human HepG2 hepatocyte cell line and found that the promoter regions of *Igf1*-targeting miRNAs (including members of the let-7 family and miR-122) are enriched for EZH2 and H3K27me3, and are frequently found in CpG-rich chromatin regions (Figure 2I). These results suggest that many of the miRNAs that target *Igf1* might be regulated by polycomb group repression. Quantification of EZH2 protein levels in aged females showed decreased EZH2 expression in liver, but not muscle in *Myc* ^*+/−*^ *versus Myc* ^*+/+*^ mice (Figure 2J). Although these results point to a possible involvement of EZH2 in the regulation of miRNA genes by MYC, more work remains to be done to confirm this hypothesis.

**FIGURE 3:**
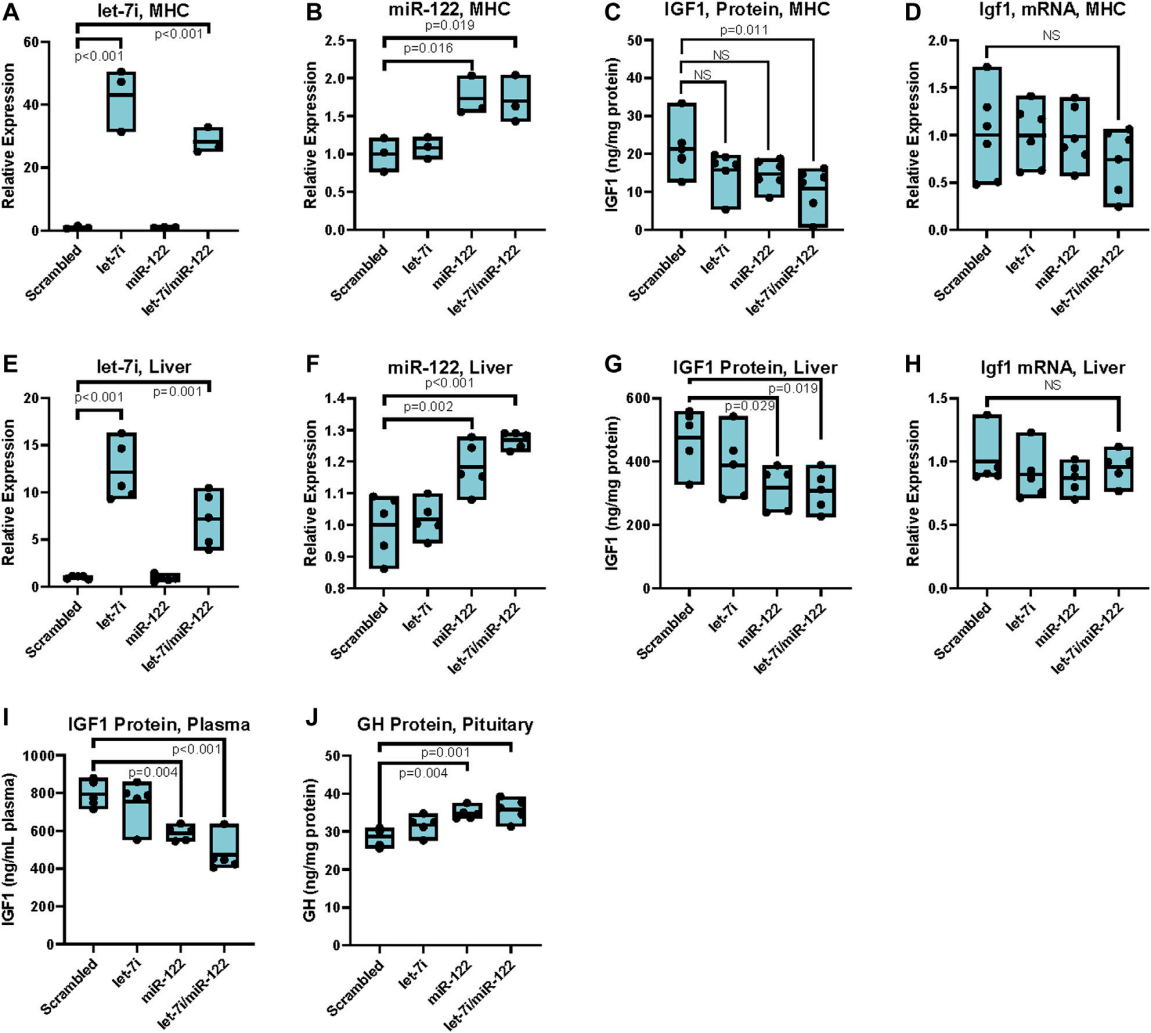
Upregulation of miR-122 inhibits IGF-1 translation *in vitro* and *in vivo* in females. **(A)** Let-7i expression in primary mouse hepatocytes (MHC) post transfection with 20 nM mimics as indicated, measured by RT-qPCR (primer pair 11), normalized to Snord 70 (primer pair 13). *n* = 3, females, 6–8 weeks. **(B)** miR-122 expression in MHC post transfection with 20 nM mimics as indicated, measured by RT-qPCR (primer pair 12), normalized to Snord 70 (primer pair 13). *n* = 3, females, 6–8 weeks. **(C)** IGF1 protein levels as assessed by ELISA in MHC) transfected with 20 nM of indicated miRNA mimics. n = 6, females, 6–8 weeks. **(D)** Igf1 mRNA levels (primer pair 7) as assessed by RT-qPCR normalized to GAPDH (primer pair 4) in primary MHC transfected with 20 nM of indicated miRNA mimics. *n* = 6, females, 6–8 weeks. **(E)** Let-7i expression in liver tissue of mice injected with 1 nmol indicated miRNA mimics via the tail vein, measured by RT-qPCR (primer pair 11), normalized to Snord 70 (primer pair 13). *n* = 5, 3 months old, females. **(F)** miR-122 expression in liver tissue of mice injected with 1 nmol indicated miRNA mimics via the tail vein, measured by RT-qPCR (primer pair 12), normalized to Snord 70 (primer pair 13). *n* = 5, 3 months old, females. **(G)** IGF1 protein levels as assessed by ELISA normalized to total protein in liver tissue of mice injected with 1 nmol indicated miRNA mimics via the tail vein. *n* = 5, 3 months old, females. **(H)** *Igf1* mRNA levels (primer pair 7) as assessed by RT-qPCR normalized to GAPDH (primer pair 4) in liver tissue of mice injected with 1 nmol indicated miRNA mimics via the tail vein. *n* = 5, 3 months old, females. **(I)** IGF1 protein levels as assessed by ELISA in plasma of mice injected with 1 nmol indicated miRNA mimics via the tail vein. *n* = 5, 3 months old, females. **(J)** GH protein levels as assessed by ELISA in pituitary extracts of mice injected with 1 nmol indicated miRNA mimics via the tail vein. n = 5, 3 months old, females. Statistical significance was computed using one-way ANOVA followed by Dunnett’s *post hoc* test. Bars represent mean, minimum, and maximum values.

To further elucidate the effects of upregulating miR-122 and let-7 in the liver, we transfected LNA-modified miRNA mimics of these miRNAs, both alone and in combination, into primary hepatocytes isolated from wild type C57Bl/6 mice. Transfection resulted in a significant intracellular increase in both let-7i and miR-122 as measured by RT-qPCR. Specifically, transfection with 20 nM miRNA mimics resulted in a 30 to 40-fold increase in let-7i (Figure 3A). Transfection with miR-122 increased its expression to 1.7-fold over scrambled control, though due to the very high expression of miR-122 in hepatocytes, this increase translates to a significant upregulation of the miR-122 miRNA (Figure 3B). Transfection with let-7i or miR-122 decreased IGF1 protein levels by ∼20% but did not achieve significance, while transfection with both let-7i and miR-122 in combination significantly decreased IGF1 protein levels by ∼40% relative to scrambled control (Figure 3C). Transfection with let-7i, miR-122, or let-7i/miR-122 combined did not affect *Igf1* transcript levels as assessed by RT-qPCR in primary hepatocytes (Figure 3D). These results are consistent with our *in vivo* data in *Myc* ^*+/−*^ vs. *Myc* ^*+/+*^ female mice which showed a decrease in IGF1 protein, but not transcript, levels (Figures 1A–C). Together, these results show that upregulation of let-7i and miR-122 can decrease IGF1 protein levels while not significantly affecting *Igf1* mRNA levels.

To assess whether upregulation of let-7 or miR-122 *in vivo* can mediate translational repression of *Igf1* we injected wild-type C57Bl/6 mice with 1 nmol of each miRNA either alone or in combination by tail-vein injection using invivofectamine as a carrier. Tissues were harvested at 4 days post-injection, with retrograde perfusion of the liver prior to harvest. Injection of 1 nmol of let-7i increased its expression 12-fold relative to scrambled control, while injection of the same amount of miR-122 increased its expression 1.2-fold (Figures 3E, F). In line with our data in primary hepatocytes, upregulation of let-7i alone resulted in a slight decrease in IGF1 protein levels which did not achieve statistical significance. Compared to scrambled control, injection of miR-122 either alone or in conjunction with let-7i significantly reduced IGF1 protein levels by almost 50% in the liver, without significantly affecting *Igf1* transcript levels (Figures 3G, H). Furthermore, treatment of mice with miR-122 or combined let-7i/miR-122 significantly decreased plasma levels of IGF1 (Figure 3I). In turn, growth hormone levels were increased in the pituitary, in line with the known negative-feedback loop between these two hormones (Figure 3J).

While decreased IGF1 levels have been associated with longevity in numerous model organisms, as well as in humans, decreased IGF1 expression with age has also been correlated with increased risk of osteoporosis, muscle-wasting, and dementia (Obermayr et al., 2005; Perrini et al., 2010; Westwood et al., 2014). Several mouse models with decreased growth hormone (and consequently IGF1) levels have been shown to have decreased bone mineral density, as well as increased trabecular spacing in old age (Palmer et al., 2009; Yakar Isaksson, 2015). Interestingly, mice with a liver-specific *Igf1* deletion that was essentially complete by 10 days of age did not show decreased femoral or body length (Sjogren et al., 1999; Yakar et al., 1999), suggesting that local, rather than endocrine, levels of IGF1 may be more important for the preservation of bone health into old age. In contrast to this data, *Igf1* liver-specific deletion at 1 year of age reduced circulating IGF1 levels by 70% and significantly reduced trabecular number, but not bone density (Gong et al., 2014). Together, these results suggest that the decrease of circulating IGF1 levels with age, rather than decreased lifetime levels of IGF1, may be responsible for age-related bone loss while low levels of circulating IGF1 from birth do not significantly impair skeletal health.

**FIGURE 4:**
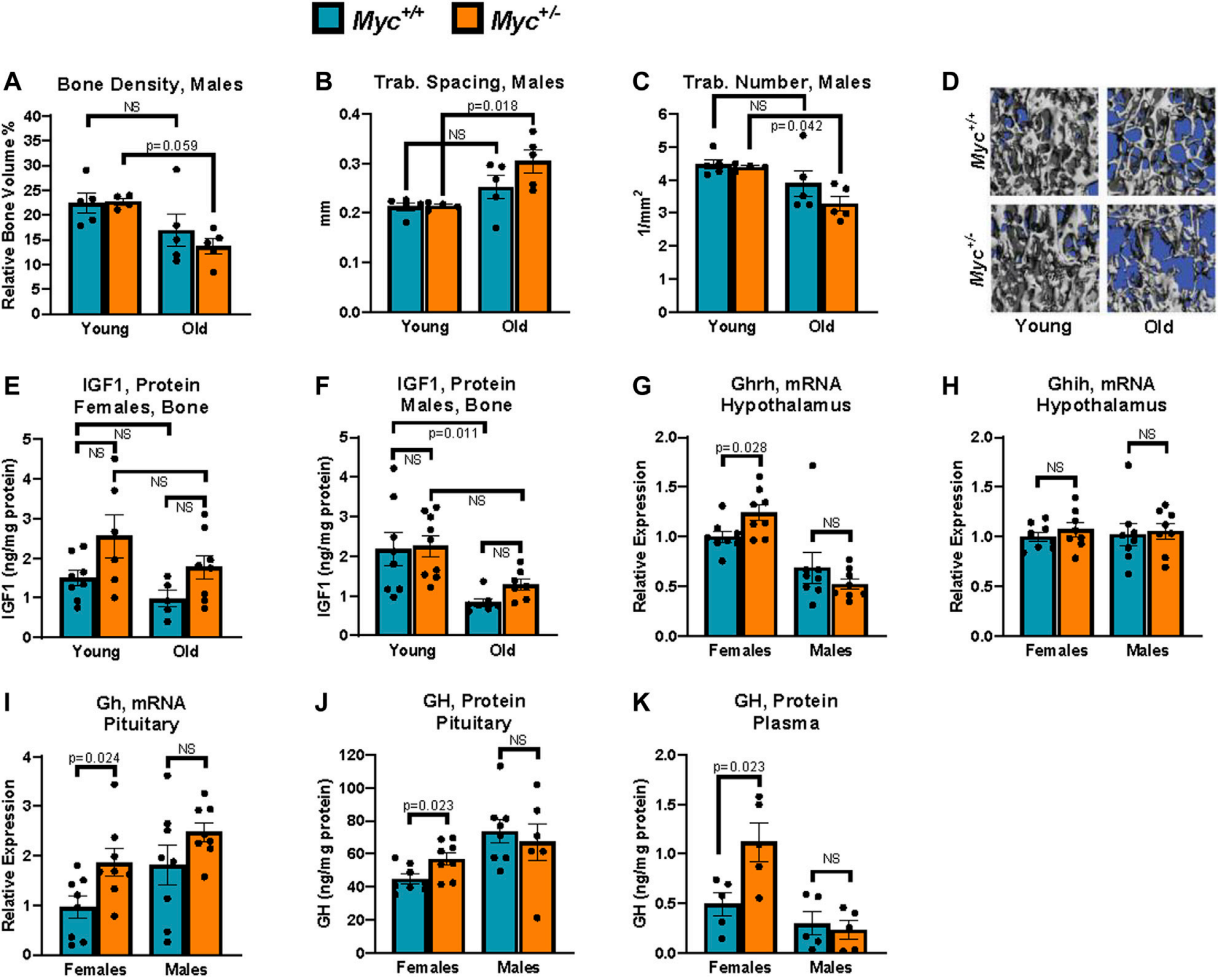
Growth hormone is upregulated through known negative-feedback loops by reduced liver IGF1 in *Myc* ^*+/−*^ relative to *Myc* ^*+/+*^ mice. **(A)** Bone density in young and old male mice measured by micro-CT. *n* = 4–5, 3 and 24 months. **(B)** Trabecular spacing (mm) in young and old male mice measured by micro-CT. *n* = 4–5, 3 and 24 months. **(C)** Trabecular number (per mm^2^) in young and old male mice measured by micro-CT. *n* = 4–5, 3 and 24 months. **(D)** Representative images used for micro-CT analysis in panels **(B, C, E)** Femur IGF1 protein levels assayed by ELISA in young and old females. *n* = 5–8, 3 and 24 months. **(F)** Femur IGF1 protein levels assayed by ELISA in young and old males. *n* = 7–9, 3 and 24 months. **(G)** *Ghrh* mRNA levels (primer pair 10) assayed by RT-qPCR normalized to GAPDH (primer pair 4) in the hypothalamus. *n* = 8, 3 months, males and females. **(H)** *Ghih* mRNA levels (primer pair 9) assayed by RT-qPCR normalized to GAPDH (primer pair 4) in the hypothalamus. *n* = 8, 3 months, males and females. **(I)** *Gh* mRNA levels (primer pair 8) assayed by RT-qPCR normalized to GAPDH (primer pair 4) in the hypothalamus. *n* = 8, 3 months, males and females. **(J)** GH protein levels assayed by ELISA in the pituitary. *n* = 6–8, 3 months, males and females. **(K)** GH protein levels assayed by ELISA in plasma. Three blood samples harvested 48 h apart were averaged for each animal. Readings greater than 3 standard deviations higher than average were discarded. *n* = 5, 4 months, males and females. Statistical significance was computed by Student’s *t*-test **(A–C and G–K)**, and one-way ANOVA followed by Tukey’s *post hoc* test **(E, F)**. Error bars represent SEM.

Female *Myc* ^*+/−*^ mice, with decreased IGF1 at all ages, show a remarkable resistance to age-related bone loss, and in fact show no decrease in bone density, trabecular number, or trabecular spacing at 22 months of age compared to *Myc* ^*+/+*^ mice (Hofmann et al., 2015). Though osteoporosis is a disease which typically affects females, we nevertheless extended our analysis to include a measurement of these parameters in male *Myc* ^*+/−*^ mice. While micro-CT analysis of the L4 vertebrae in wild-type male mice showed small trends for a decrease in bone density, increase in trabecular spacing, and decrease in trabecular number, none reached significance (Figures 4A–D). In contrast, these changes were exacerbated male *Myc* ^*+/−*^ mice and reached significance in two out of three parameters. Thus, while reduced levels of MYC positively impact skeletal health with age in females, the opposite was true for males.

To assess whether local levels of IGF1 in bone tissue were affected by *Myc* heterozygosity, we extracted protein from femurs of male and female *Myc* ^*+/+*^ and *Myc* ^*+/−*^ mice at 3 and 24 months of age. Interestingly, in both sexes, *Myc* ^*+/−*^ mice IGF1 levels in the bone tended to be higher, as opposed to the decreases we found in the liver (above), although these trends were not significant (Figures 4E, F). The lack of decreased IGF1 expression in *Myc* ^*+/−*^ mouse bone tissue suggests that MYC regulation of IGF1 is liver-specific. However, we found a significant decrease of IGF1 protein levels in males (Figure 4F), which might explain the decrease in bone health.

Mice with liver-specific ablation of *Igf1,* resulting in greater than 70% reduction in circulating IGF1, show significantly increased levels of circulating growth hormone (Gong et al., 2014). Although the details of this negative feedback mechanism remain unclear, it has been reported that both growth hormone releasing hormone (GHRH) and growth hormone inhibiting hormone (GHIH) are regulated by IGF1 levels (Obal et al., 1991; Romero et al., 2012). We thus assessed the expression of these two factors in the hypothalamus at the mRNA level, and found a modest but significant increase in *Ghrh* in the hypothalamus of female, but not male, *Myc* ^*+/−*^ mice (Figure 4G), whereas *Ghih* levels were unaffected (Fig. 7H). Consistent with this, transcript and as well as protein levels of growth hormone in the pituitary were significantly increased in female but not male *Myc* ^*+/−*^ mice (Figures 4I, J).

Circulating growth hormone levels are difficult to assess *in vivo* due to their circadian as well as feeding-dependent fluctuations, the pulsatile, time of day-dependent fluctuations, which can range by as much as 100-fold in a matter of hours. However, through repeated measurement at consistent timepoints, it is possible to determine an average baseline level for each animal (Steyn et al., 2011). We thus collected blood via the saphenous vein at 10 a.m. every other day for a total of 3 time points from each animal and GH concentrations in each sample were measured by ELISA. We found that average growth hormone levels were significantly increased in female, but not male, *Myc* ^*+/−*^ mice (Figure 4K). These results indicate that decreased liver production of IGF1 in female *Myc* ^*+/−*^ mice, and thus reduced circulating IGF1, through a negative feedback loop increase GHRH levels in the hypothalamus, and consequently increase growth hormone production and secretion by the pituitary.

## Discussion

*Myc* ^*+/−*^ mice show global upregulation of miRNA expression, in line with other reports showing that MYC represses most of its target miRNA genes. Many of these miRNAs have been implicated in various human diseases, including Alzheimer’s, osteoporosis, muscle wasting, and hepatocellular carcinoma. As upregulation of MYC has been associated with more than 50% of all tumors, as well as other aging-related diseases, the modulation of miRNA expression by MYC is likely a significant mechanism by which MYC dysregulation leads to impaired human health (Levens, 2010). Interestingly, caloric restriction has also been shown to globally increase miRNA expression, suggesting that miRNAs may play a significant role in the regulation of lifespan (Zhang et al., 2019). Here, we have shown that downregulation of MYC in female mice induces the expression of multiple miRNAs that target the *Igf1* transcript, and that at least one of these, miR-122, significantly reduces IGF1 translation upon ectopic upregulation *in vivo*.

While the mechanisms by which MYC affects transcriptional activation are well characterized, those mediating its role in transcriptional repression are more poorly understood. Several hypotheses have been proposed and supported by experimental results, including that MYC can upregulate the expression of some transcriptional repressors (Philipp et al., 1994; Lee et al., 1996), that MYC is recruited to the promoters of its repression targets through protein-protein interactions with transcriptional regulators such as TFII-I, YY-I, Sp-I, and MIZ-1 (Schneider et al., 1997; Seoane et al., 2001), and that MYC can regulate the expression of chromatin silencing factors such as the Polycomb Repressive Complex (PRC) (Knoepfler et al., 2006).

MYC has been found to regulate both the transcription of *Ezh2* and EZH2 protein activity through phosphorylation (Bhandari et al., 2011; Neri et al., 2012), but the genes that are regulated through this PcG mechanism are not fully characterized. In line with MYC’s role as a chromatin regulator, genes repressed by MYC show considerable overlap with PcG and HDAC repressed genes (Kaur and Cole, 2013; Bhadury et al., 2014). Furthermore, knockdown of *Ezh2* in glioma cells was found to upregulate 85 miRNA genes, many of which are also known to be repressed by MYC (Wang et al., 2013). In fact, many of the miRNAs upregulated in *Myc* ^*+/−*^ mice have already been documented to be regulated by EZH2 (So et al., 2011; Liu et al., 2013; Vella et al., 2015).

We found that, consistent with literature showing that MYC overexpression upregulates EZH2, female *Myc* ^*+/−*^ mice showed reduced liver EZH2 protein levels compared to *Myc* ^*+/+*^ mice. Analysis of available Encode ChIP-Seq datasets from the human HepG2 hepatocyte cell line showed that the promoter regions of *Igf1*-targeting miRNAs, including most members of the let-7 family and miR-122, are enriched for EZH2 and H3K27me3. These results suggest that many of the miRNAs that target *Igf1* may be regulated by PcG repression, which is regulated by MYC.

While EZH2-mediated repression is a plausible mechanism for MYC-mediated repression of miRNA genes, it was unclear why these miRNA genes were upregulated specifically in female *Myc* ^*+/−*^ mice. Many miRNA genes have been shown to be regulated in a sexually dimorphic manner. For example, in rat liver the IGF1-targeting miR-193a, miR-29b, and miR-122 miRNAs were found to be expressed at higher levels in females relative to males (Cheung et al., 2009). Another group found that 37% of miRNA genes were differentially expressed when MCF-7 human breast cancer cells were exposed to estrogen (Hah et al., 2011). These results were corroborated by another group which showed that estrogen induces the expression of 21 miRNAs while down-regulating 7 in MCF-7 cells (Bhat-Nakshatri et al., 2009). The miRNAs upregulated by estrogen in these cell types (the let-7, miR-30, miR-23 families), also overlap extensively with the miRNAs that target the *Igf1* transcript, as well as those upregulated in *Myc* ^*+/−*^ females. Interestingly, it has been shown that EZH2 can be recruited to target gene promoters through estrogen receptor alpha, and that testosterone administration to female mice, which downregulates ER-alpha expression, results in the upregulation of several miRNAs, including members of the let-7 family, miR-122, and miR-30d (Delic et al., 2010; Ariazi et al., 2017).

Estrogen also plays a significant role in the regulation of MYC targets, and miR-122 expression has been previously shown to be upregulated in females compared to males (Cheung et al., 2009). miR-122 is the predominant miRNA expressed in liver tissue, compromising more than half of the total miRNA pool in the liver, according to our analyses and those of others (Jopling, 2012). miR-122 is regulated by multiple liver-enriched transcription factors, including HNF3b (FOXA2), HNF4a, and HNF6 (Xu et al., 2010). The miR-122 regulating liver-enriched transcription factor FOXA1/2 alternatively regulates MYC depending on the presence of estrogen or androgen (Li et al., 2012). This sex-hormone specific regulation is believed to be an important contributor to the sexual dimorphism of hepatocellular carcinoma, which has a prevalence in males 2–4 times higher than in females (Hsu et al., 2012; Li et al., 2012). In fact, downregulation of several oncogenic genes by FOXA1/2 is dependent on the presence of estrogen (Li et al., 2012). While estrogen-dependent regulation of miR-122 by FOXA1/2 has not been explicitly described, it is a plausible explanation for the sex-specific upregulation of miR-122 in *Myc* ^*+/−*^ female mice.

Of the many miRNAs that are predicted to target the *Igf1* transcript, our results showed that miR-122 had the most profound effects on IGF1 translation in *Myc* ^*+/−*^ female mice. Given that miR-122 is a liver-specific miRNA, this suggests that MYC regulates IGF1 in a liver-specific manner. In line with this, we found that female *Myc* ^*+/−*^ mice did not have decreased IGF1 levels in bone tissue, in contrast to the significant reduction seen in the liver. In fact, IGF1 levels in femur tissue of *Myc* ^*+/−*^ mice trended towards increased expression both at young and old age.

IGF1 and its main regulator, growth hormone, function in a negative feedback loop such that reduced IGF1 levels in the circulation trigger an increase in growth hormone levels in the pituitary, and its secretion into circulation (Romero et al., 2012). We found increased GH expression and circulating levels in *Myc* ^*+/−*^ female mice, which is likely explained by the known negative-feedback loop between circulating IGF1 and GH. Increased growth hormone in circulation of *Myc* ^*+/−*^ female mice may then signal to peripheral tissues such as the bone to stimulate local production of IGF1, thereby mitigating bone loss with age, and possibly explaining the remarkable resistance of *Myc* ^*+/−*^ female mice to age-related bone loss.

Together, these results suggest that liver-specific reduction in IGF1 does not affect local production of IGF1 in peripheral tissues, and that in fact it may enhance it. Several mouse models with liver-specific disruption of IGF1 have been generated. Constitutive liver-specific *Igf1* knockout resulted in a >60% reduction in circulating IGF1 levels and had minimal effects on body weight, organ weight, and femoral length (Yakar et al., 1999). Liver-specific ablation of IGF1 at 3 weeks of age resulted in a ∼75% decrease in circulating IGF1 levels, extended lifespan by 16% in females, but resulted in only minor reduction in femoral length (Sjogren et al., 1999; Svensson et al., 2011). Unfortunately, local IGF1 levels were not measured in bone tissue of either mouse model, nor was trabecular morphology assessed with age. However, the results of these studies are consistent with our data, and suggest that liver-specific disruption of IGF1 at young age does not significantly affect bone health.

In aggregate, we propose a model in which MYC-mediated activation of EZH2 causes the upregulation of multiple miRNAs (namely, miR-29, let-7, and miR-122), in an estrogen-dependent manner, which then target the *Igf1* transcript and reduce its translation. The regulation of *Igf1* translation by miR-122, which is a liver-specific miRNA that showed the most pronounced effect on *Igf1* translation, results in the reduction of IGF1 protein levels in the liver of female *Myc* ^*+/−*^ mice. Since circulating IGF1 is produced predominantly in the liver, the systemic effect of decreased MYC expression is reduced plasma levels of IGF1. Given that IGF1 and GH function in a negative feedback loop, such that reduced circulating IGF1 triggers an increased production and secretion of GH by the pituitary, decreased MYC expression concomitantly increases plasma levels of GH. Increased GH expression, in turn, increases the local production of IGF1 in tissues such as the bone, thereby evading some of the negative consequences of global IGF1 reduction.

## Data Availability

The AGO-CLIP-seq data presented in the study are deposited in the NCBI Sequence Read Archive (SRA) BioProject repository, accession number PRJNA1004998.
