# Supplementary material for: Regulation of the somatotropic axis by MYC-mediated miRNA repression
Source: Front Cell Dev Biol. 2023 Oct 16;11:1269860. doi: 10.3389/fcell.2023.1269860 (PMC10615138; doi:10.3389/fcell.2023.1269860)
Supplement: Supplementary file 1 [file Table1.DOCX]

**List of primers used in PCR analysis**

| **Primer #** | **Name** | **Sequence** | **Purpose** |
| --- | --- | --- | --- |
| Primer pair 1 | MYCFLXS | GCCCCTGAATTGCTAGGAAGACTG | Intron 1 of Myc, 5' of the 5' Lox site, sense orientation. |
|  | MYCFLXA | CCGACCGGGTCCGAGTCCCTATT | Intron 1 of Myc, 3' of the 5' Lox site, antisense orientation. |
|  |  |  | Used for genotyping. Primer pair will amplify 378 bp fragment from Myc+/+ (wild-type) chromosome, and nothing from the Myc+/– (deletion) chromosome, because primers flank 5' Lox site. |
| Primer pair 2 | MYCDELS | TCGCGCCCCTGAATTGCTAGGAA | Intron 1 of Myc, 5' of the 5' Lox site, sense orientation. |
|  | MYCDELA | TGCCCAGATAGGGAGCTGTGATACTT | Used for genotyping. Downstream of Myc, 3' of the 3' Lox site, antisense orientation. Primer pair will amplify ~450 bp fragment from Myc+/– (deletion) chromosome, and nothing from the Myc+/+ (wild-type) chromosome, because the primers are too far apart. |
| Primer pair 3 | XZMCMYC1F | TTCCTTTGGGCGTTGGAAAC | At the 3' end of Myc exon 1, sense orientation. |
|  | XZMCMYC1R | GCTGTACGGAGTCGTAGTCG | In Myc exon 2, antisense orientation. |
|  |  |  | Used for RT-qPCR of Myc mRNA. Primers span intron 1. |
| Primer pair 4 | MGAPDHF | CGGCCGCATCTTCTTGTG | Sense orientation. |
|  | MGAPDHR | GTGACCAGGCGCCCAATA | Antisense orientation. |
|  |  |  | Used for RT-qPCR of the mRNA of the Gapdh gene. Primers span an intron. |
| Primer pair 5 | APBP3F | AACCTGCTCCAGGAAACATCA | Sense orientation. |
|  | APBP3R | AACTTGGAATCGGTCACTCGG | Antisense orientation. |
|  |  |  | Used for RT-qPCR of the mRNA of the Igfbp3 gene. Primers span an intron. |
| Primer pair 6 | APIGF1RF | GCTTCGTTATCCACGACGATG | Sense orientation. |
|  | APIGF1RR | GAATGGCGGATCTTCACGTAG | Antisense orientation. |
|  |  |  | Used for RT-qPCR of the mRNA of the Igf1r gene. Primers span an intron. |
| Primer pair 7 | APIGF34F | GCTGGTGGATGCTCTTCAGT | Sense orientation. |
|  | APIGF34R | TCCGGAAGCAACACTCATCC | Antisense orientation. |
|  |  |  | Used for RT-qPCR of the mRNA of the Igf1 gene. Primers span exons 3 and 4, which are present in all Igf1 isoforms. |
| Primer pair 8 | APGHF | CCTGTGGACAGATCACTGCTT | Sense orientation. |
|  | APGHR | CAGCCATGACTGGATGAGCA | Antisense orientation. |
|  |  |  | Used for RT-qPCR of the mRNA of the Gh gene. Primers span an intron. |
| Primer pair 9 | APGHIHF | AAGATGCTGTCCTGCCGTC | Sense orientation. |
|  | APGHIHR | CCAGTTCCTGTTTCCCGGTG | Antisense orientation. |
|  |  |  | Used for RT-qPCR of the mRNA of the Ghih gene. Primers span an intron. |
| Primer pair 10 | APGHRHF | CAGGATGCAGCGACACGTAG | Sense orientation. |
|  |  | GGAATCCCTGCAAGATGCTCTC | Antisense orientation. |
|  | APGHRHR |  | Used for RT-qPCR of the mRNA of the Ghrh gene. Primers span an intron. |
| Primer pair 11 | APLET7IF | AGTGAGGTAGTAGTTTGTGCT | Sense orientation. |
|  | APLET7IR | GGTCCAGTTTTTTTTTTTTTTTAACAG | Antisense orientation. |
|  |  |  | Used for RT-qPCR of the mature let-7i miRNA. |
| Primer pair 12 | APMIR122F | GCAGTGGAGTGTGACAATG | Sense orientation. |
|  | APMIR122R | CCAGTTTTTTTTTTTTTTTCAAACACC | Antisense orientation. |
|  |  |  | Used for RT-qPCR of the mature miR-122 miRNA. |
| Primer pair 13 | JKSNORD70F | TGGAACTGAATCTAAGTGATTTAACAAA | Sense orientation. |
|  | JKSNORD70R | CCAGTTTTTTTTTTTTTTTCTCAGTG | Antisense orientation. |
|  |  |  | Used for RT-qPCR of the mRNA of Snord70. |
| Primer pair 14 | APDP3Y | GTCTCGTGGGCTCGGAGATGTGTATAAGAGACAGAGGGAGGACGATGCGG | Sense orientation. |
|  | APDP5Y | TCGTCGGCAGCGTCAGATGTGTATAAGAGACAGCCGCTGGAAGTGACTGACAC | Antisense orientation. |
|  |  |  | Used for PCR amplification of AGO-CLIP-Seq tags. |
| Primer pair 15 | APTSP5 | AATGATACGGCGACCACCGAGATCTACACTAGATCGCTCGTCGGCAGCGTC | Sense orientation. |
|  | APTSSP7.1 | CAAGCAGAAGACGGCATACGAGATTCGCCTTAGTCTCGTGGGCTCGG | Antisense orientation. |
|  |  |  | Used for addition of sequencing indeces to AGO-CLIP-Seq tags. |
| Primer pair 16 | APTSP5 | AATGATACGGCGACCACCGAGATCTACACTAGATCGCTCGTCGGCAGCGTC | Sense orientation. |
|  | APTSSP7.2 | CAAGCAGAAGACGGCATACGAGATCTAGTACGGTCTCGTGGGCTCGG | Antisense orientation. |
|  |  |  | Used for addition of sequencing indeces to AGO-CLIP-Seq tags. |
| Primer pair 17 | APTSP5 | AATGATACGGCGACCACCGAGATCTACACTAGATCGCTCGTCGGCAGCGTC | Sense orientation. |
|  | APTSSP7.3 | CAAGCAGAAGACGGCATACGAGATTTCTGCCTGTCTCGTGGGCTCGG | Antisense orientation. |
|  |  |  | Used for addition of sequencing indeces to AGO-CLIP-Seq tags. |
| Primer pair 18 | APTSP5 | AATGATACGGCGACCACCGAGATCTACACTAGATCGCTCGTCGGCAGCGTC | Sense orientation. |
|  | APTSSP7.4 | CAAGCAGAAGACGGCATACGAGATGCTCAGGAGTCTCGTGGGCTCGG | Antisense orientation. |
|  |  |  | Used for addition of sequencing indeces to AGO-CLIP-Seq tags. |
| Primer pair 19 | APTSP5 | AATGATACGGCGACCACCGAGATCTACACTAGATCGCTCGTCGGCAGCGTC | Sense orientation. |
|  | APTSSP7.5 | CAAGCAGAAGACGGCATACGAGATAGGAGTCCGTCTCGTGGGCTCGG | Antisense orientation. |
|  |  |  | Used for addition of sequencing indeces to AGO-CLIP-Seq tags. |
| Primer pair 20 | APTSP5 | AATGATACGGCGACCACCGAGATCTACACTAGATCGCTCGTCGGCAGCGTC | Sense orientation. |
|  | APTSSP7.6 | CAAGCAGAAGACGGCATACGAGATCATGCCTAGTCTCGTGGGCTCGG | Antisense orientation. |
|  |  |  | Used for addition of sequencing indeces to AGO-CLIP-Seq tags. |
| Primer pair 21 | APTSP5 | AATGATACGGCGACCACCGAGATCTACACTAGATCGCTCGTCGGCAGCGTC | Sense orientation. |
|  | APTSSP7.7 | CAAGCAGAAGACGGCATACGAGATGTAGAGAGGTCTCGTGGGCTCGG | Antisense orientation. |
|  |  |  | Used for addition of sequencing indeces to AGO-CLIP-Seq tags. |
| Primer pair 22 | APTSP5 | AATGATACGGCGACCACCGAGATCTACACTAGATCGCTCGTCGGCAGCGTC | Sense orientation. |
|  | APTSSP7.8 | CAAGCAGAAGACGGCATACGAGATCCTCTCTGGTCTCGTGGGCTCGG | Antisense orientation. |
|  |  |  | Used for addition of sequencing indeces to AGO-CLIP-Seq tags. |

1All sequences are listed in the 5'->3' orientation.

2These primers pairs were used for genotyping of animals using tail snip DNA.

Both primer pairs were included in the same PCR reaction.
